# Impact of Soil Pollution on Melliferous Plants

**DOI:** 10.3390/toxics10050239

**Published:** 2022-05-09

**Authors:** Alina Bărbulescu, Lucica Barbeș, Cristian Ştefan Dumitriu

**Affiliations:** 1 Department of Civil Engineering, Transilvania University of Brașov, 5 Turnului Str., 900152 Brasov, Romania; alina.barbulescu@unitbv.ro; 2 Department of Chemistry and Chemical Engineering, Ovidius University of Constanța, 124 Mamaia Bd., 900527 Constanta, Romania; 3 S.C. Utilnavorep S.A., 55 Aurel Vlaicu Av., 900055 Constanta, Romania

**Keywords:** pollution indices, *Sambucus nigra* L., *Hypericum perforatum*, *Tilia tomentosa*, statistical analysis

## Abstract

This study aims at providing bee products and derivatives of medicinal plant consumers with a multifaceted perspective on mineral elements occurring in the soils of two forest zones in the vicinity of North Dobrogea (Romania) by (1) analyzing the pollution levels of the soils at three sites (denoted by DS, PH, and ST) in the study region, using different indicators; (2) providing the results of the transfer of metals from the soil to *Sambucus nigra* L. (*SnL*), *Hypericum perforatum* (*Hp*), and *Tilia tomentosa* (*Tt*). The statistical analysis of the series collected at these locations shows no difference between the elements’ concentrations (as a whole). Still, the values of the geo-accumulation index (*I_geo_*) classify the soils as being soils that are moderately to highly contaminated with Cd (and not contaminated with Cu, Mn, or Zn) with respect to the European background values. The cumulative indices—the degree of contamination (*DC*), the pollution load index (*PLI*), the Nemerow integrated pollution index (*NIPI*)*,* and the potential ecological risk index (*PERI*) indicated the highest contamination in DS (which is a tourist area). To assess the accumulation of different metals in plants, the enrichment factors (*EF*) were computed. In over 75% of cases, *EF* was above 1, indicating a high degree of enrichment with different metals. The highest values were those for Cu (41.10 in DS for *SnL*), and Cd (12.85 in DS for *Tt*). The results showed that there were different degrees of accumulation between microelements and trace elements in the plants. *Tt* acted as a bioaccumulator for almost all of the studied elements (K, Mg, Na, Fe, Mn, Cu, Zn, and Cd).

## 1. Introduction

Melliferous plants are the primary food source for bees, which provide valuable biological products, such as honey, pollen, nectar, propolis, tea infusions, and other food/cosmetics products [1,2]. The productivity of bee families and their honey quality depend directly on the natural environmental conditions. These relate to the floristic composition of honey plant species, and the climatic, pedological, and phenological factors [3,4,5]. Due to the existing pedoclimatic conditions, Romania has good conditions for beekeeping and apiculture development. Romania’s honey flora includes approximately 220 species [6]. For the composition of honey varieties, these mainly consist of carbohydrates (glucose and fructose from 65 to 80% of the total soluble solids), which are directly assimilable by human organisms [7]. Along with the two primary components, honey contains a wide range of nutrients, such as minerals, vitamins, organic acids, aromatic substances, or compounds with an antibiotic role, borrowing from the properties of the plants of origin [5,8]. Lazarevic et al. [7] show that the mineral content of honey is relatively low (in nectar, it is from 0.1 to 0.2%, and in honeydew, it is over 1%). Potassium is the most abundant metal in this bee product, comprising 45–85% of the total mineral content [7]. Barbes et al. [9] measured the maximum concentrations of macroelements in different bee product samples (K—685.2 mg/kg in multi-floral honey, and Ca—42.65 mg/kg in the honeycomb). Additionally, the authors reported a maximum concentration of Zn (3.05 mg/kg in propolis), Fe (1.22 mg/kg in honeycomb), Cr (0.08 mg/kg in lime honey obtained from the forest zone, and 7.24 mg/kg in acacia honey from an agricultural location), and Pb (0.671 mg/kg in multi-floral honey from a polluted area) [9]. Thus, the mineral content from different bee products is closely related to the botanical species and its geographical origins, which depends on the floristic type of the melliferous plant and the soil composition. Barbes et al. [10] indicated the mean content of macroelements (Ca—4.832 g/kg d.w. in *Tilia tomentosa* (*Tt*), K—45.52 g/kg d.w. in *Sambucus nigra* L. (*SnL*)), microelements, and trace metals (Fe—185.83 mg/kg d.w. in *Hypericum perforatum* (*Hp*), Mn—35.74 mg/kg d.w. in *SnL*, Cu—43.62 mg/kg d.w. in *Hp*, Zn—38.6 mg/kg *SnL*, Cd—5.07 mg/kg d.w. in *Tt*) that are present in some wild plant flowers with the highest melliferous potential, that were harvested from forest areas and other nearby areas adjacent to heavy road traffic. 

The soil is an essential factor in plant nutrition. It is also an open system, the activity of which depends mainly on the energy flow that is received from the sun through the vegetation that is present on its surface. The effects of pollution on soils changes their chemical composition (including metal species) and some essential characteristics, such as pH, microbiological activity, or humidification processes, producing severe consequences for soil quality. The quantitative monitoring of chemical species in the soil is necessary for all elements that could be sources of contamination for the soil and vegetation [11,12]. If air or water can return to a similar composition as that of the initial state by applying adequate measures to eliminate pollution, it is very different in the case of soil, which is a more complex part of the environment and whose compounds are in a balance that has been achieved over a long period of time [13]. Some metal species (Cu, Zn, Mn, Fe, etc.) are natural constituents that are essential to plant development [14]. However, other metals, such as Ni, As, Cr, or Pb, can be toxic to plants, even at low concentrations [15]. Additionally, critical metal species, when present at high concentrations, can have harmful effects on plants or other ecosystems [12,14]. 

One of the fastest ways to transfer metal species to the human body is via the consumption of products of plant origin (medicinal plants) or derived origin (bee products), resulting from a contaminated area [16]. This transfer route is of particular interest if the products come from a source that is located in a polluted area. Some studies indicate that pH variation and carbonate dissolution can produce the accumulation of Cu, Mn, and Pb in plant leaves. Soil acidification (pH changes) in the root zone leads to increased Zn and Cu mobility. An increase in Cd mobility seems to result from a decrease in pH [17,18]. It was shown [14,19] that the appearance of ammonium ions is responsible for pH variations over time for non-hyperaccumulator plants, while for hyperaccumulators, other processes are specific. Several plant-specific mechanisms, such as respiration and transpiration, sorption, dissolution, precipitation, and redox reactions may cause changes in the chemical conditions of the rhizosphere (the interface between the roots and the soil). Some products that generate various complexes with metal ions are eliminated from the plant roots in the rhizosphere [20]. These products are oxalic, acetic, citric, tartaric, uronic, and polysaccharide acid complexes. Additionally, the mechanisms that are involved in changing the rhizosphere’s chemical conditions and in taking over chemical elements depend on the species types and the soil conditions [21]. 

Plant growth involves structural modifications that are mainly a result of climate change caused by anthropogenic activities, especially over the last few decades. Thus, various metal species in the vegetation have become a global problem, due to the intensification of forestry and mining. As a result of these exploitations, soil degradation frequently occurs, and is the worst form of pollution because it expands in large areas [22,23,24,25]. Anthropogenic activities have led to significant augmentations of the concentrations of metal species in the soil, especially in areas with heavy traffic [26,27,28,29]. Yang et al. [27] evaluated the concentrations of metals and the pollution levels of different samples that were collected from China. The authors reported high quantities of heavy metals resulting from the mining and processing of extracted metals. The study indicates resultant pollution with Cd, Cu, and Zn from these activities. 

The recorded concentrations of Cu, Ni, Zn, Co, Cr, Cd, and Pb from soil samples in the Romanian North Littoral have indicated increased Zn (221.28 ± 7.0 mg/kg) and Pb (25.66 ± 0.22 mg/kg) values that exceed the baseline range of 100 mg/kg and 20 mg/kg, respectively [29]. The soil samples were taken from near industrial areas and high traffic density areas from May to October 2010. Additionally, the bioaccumulation of the metals in vegetation (*Taraxacum officinale* L. and *Populus nigra* L.) increased over the dry period [28,29]. The concentrations of Pb, Mn, Cr, and Cd in the components of *Plantago major* and *Taraxacum officinale* L. from Ciudanovita and Lisava, and from Banat Region, Romania, were above the admissible limits as well [16]. These mineral elements are found mainly in metallic form in the soil, and possess low toxicity. On the other hand, if they transform into a soluble form with increased bioavailability, they become dangerous for flora, fauna, and humans. 

Pollution analysis is sometimes made using multivariate statistical methods [30,31,32,33]. Principal component analysis has been widely employed to determine the sources of soil pollution, and to differentiate between anthropic and natural contributions. It is often coupled with cluster analysis for grouping the studied characteristics [27]. To investigate the absorption of chemical elements by vegetation in contaminated sites, the bio-accumulation factor (BF) is frequently utilized [15,16]. 

There are significant differences in the chemical behavior of metal species in the soil–plant system. A helpful tool within the analysis is the use of the translocation factor. This is a tool for evaluating the translocation of different chemical elements from the plant’s roots to the shoots [15,16,17]. 

The geo-accumulation index (*I_geo_*) is utilized to quantify the interaction between toxic elements in the absorption and translocation of metals in plants, which indicates the level of pollution in the rhizosphere. Shen et al. [17] indicate synergistic interactions in the accumulation of some metals in *P. ostii*. The study site is near the tailings dump of a mining copper from Tongling, China. Additionally, the calculation of *I_geo_* for medicinal plants showed that copper and lead were the principal metals in the rhizosphere whose concentrations exceeded the maximum permissible limits for human health. The calculated translocation factor showed that *Paeonia ostii* acted as a bioaccumulator for cadmium and lead [17]. 

Based on the above, we considered it necessary to investigate pollution levels for an area located in the southeastern part of Romania, given that an enormous amount of honey results from deciduous forest vegetation mixed with numerous medicinal plants. In North Dobrogea, beekeeping has experienced fulminant developments over the last two decades. However, poorly controlled logging activities, complemented by those in stone quarries and the chaotic management of household waste in the area, have generated significant amounts of soil pollution, and large amounts of toxic pollutants have been stored over the long term. This study refers to two protected forest sites and another one in the near vicinity that is affected by heavy traffic, from the Plateau of North Dobrogea, between 2019 and 2020. The research results are new for the following reasons: (1) No studies have used statistical analysis to quantify the pollution levels in different forest zones of North Dobrogea; (2) No studies have used multivariate statistics to evaluate the efficiency of metal accumulation in melliferous plants from this areal zone; (3) No studies have used different health risk indicators to characterize the metal profiles of melliferous/medicinal plants or other derivatives products obtained from beekeeping in the North Dobrogea Plateau. 

## 2. Materials and Methods

### 2.1. Study Area

**Figure 1:**
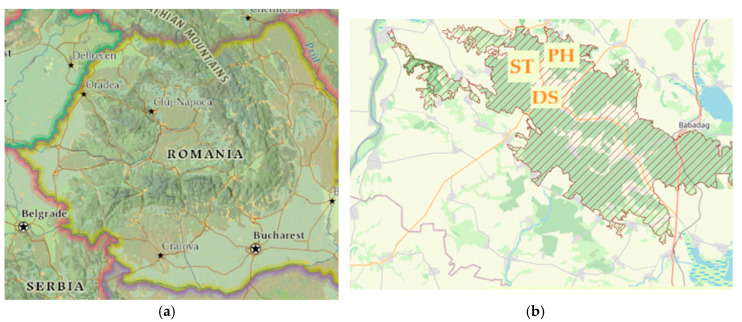
(**a**) Map of the studied locations. ES (https://www.arcgis.com/home/webmap/viewerhtml) (accessed on 20 March 2022); (**b**) ROSPA0091 Babadag Forest: PH—Peony Hill (Dealul Bujorului); ST—Secaru Mountain Top (Vârfu Secaru); DS—Deer Source (Izvorul Cerbului) (https://eunis.eea.europa.eu/sites/ROPA0091) (accessed on 20 March 2022).

The experimental study was developed near the Ciucurova forest in Romania’s North Dobrogea Plateau (Figure 1). The Ciucurova district, located on the upper course of the Slava river, has large areas with deciduous forests, rich linden resources (*Tilia* spp.), and medicinal plant species (e.g., *Hippericum* spp. and *Adoxaceae* spp.). Melliferous trees grow on land with altitudes between 70 and 400 m. The melliferous potential of this natural area is represented chiefly (45% of the total area) by linden (*Tilia* sp. on 2500 ha, 1300 ha, aged 30 years). Ciucurova village is located at an average altitude of 137 m above sea level. On the administrative territory of Ciucurova, there are two protected natural areas—Dealul Bujorului (Peony Hill—PH) and Vârful Secaru (Secaru Hill mountain top-ST) [34]. PH Nature Reserve (44°57′35″ N; 28°29′00″ E), part of the Măcinului Mountains National Park, is in the central-northern area of the Babadag Plateau, approximately 4 km N-E of Atmagea village, Ciucurova district, and it has a total area of 50.8 ha. Specific associations of the loess steppe characterize the grassy vegetation. The forest vegetation has a mosaic aspect in which the sub-Mediterranean forests intersect with the forest-steppe woodlands. The ST Nature Reserve (44°57′14″ N; 28°24′19″ E) is situated in the western part of the Babadag Plateau, approximately 4 km southwest of Atmagea, and is 34.5 ha in area. The ST Nature Reserve is a place of granitic erosion and is the highest peak (400 m) in the Babadag Plateau. Specifically, ST looks like an island with slightly more acidophilic vegetation than the rest of the Babadag Plateau, where the calcophilic species are characteristic. In the meadows, the grassy associations of the pontic steppe predominate on superficial soils next to isolated trees or in clusters.

Due to the diversity and the quality of the products that it supplies, beekeeping represents a fast-growing and highly promising branch of these lands. The slope (slope of the land) is a physical–geographical factor with a determining ecological role for the studied areas, significantly influencing the practical physiological volume of the soils, the humidity regime, and the nutrient content. Additionally, the associations of chernozem soils, which are the most extensive, with less developed soils, occupy most of the area, favoring a structure dominated by forest lands (60%), agricultural (35%), and other uses (5%). At a depth of between 0 and 20 cm, the soil pH is between 6.87–7.2 (slightly acid to neutral soil), contains soluble salts at 20%, with TOC (g/kg) in the range of 12.08–14.65, and a TN (g/kg) between 0.87 and 0.92.

The climate of the Babadag Plateau is continental and maritime coastal. The air masses in the north and north-east lose much of their moisture over the European continent, and they end up above Dobrogea, which is poor in rainfall. The average annual temperatures highlight the existence of cold springs, sweltering summers, and thermally moderate winters. According to recorded quantities (350–510 mm), the average yearly rainfall places Dobrogea among the regions with the lowest values in Romania. There is a marked manifestation of long-term aridity. In addition to human intervention, the forests of the Babadag Plateau are not immune to certain natural, physical, or biological disasters. These include late summer drought, which limits the growth of most species. Different processes leading to landscape degradation and a reduction in its degree of naturalness has been observed. The action of external agents has generated land degradation through erosion, transport, and accumulation. In addition to natural processes caused by biological factors, the degradation of landscapes is accentuated by anthropogenic activities such as chaotic waste storage, which is a significant problem in areas with a high flow of tourists at stops at the edge of the forest area (e.g., Deer Source camping—44°55′33.5″ N; 28°26′54.1″ E). The waste quantity increases via the storage of construction materials frequently used in this area, such as stone, earth with twigs, and wood.

### 2.2. Sampling and Analysis of Melliferous Plants and Soil

The study was carried out from May 2019 to August 2019, when the melliferous plants are in the best blooming period. Samples of surface soil were collected near the crossing road of the DS site (considered to be a polluted site—the average number of circulated vehicles increased from the end of March to the middle of September from 250 per day to 1000 per day on the only single road), and from the PH areal site and the ST areal site, respectively (Figure 1). The last two sites are supposed to be less polluted. Spontaneous plants (linden—*Tilia tomentosa*—*Tt*; rattle or St John’s wort—Hypericum perforatum—*Hp*; elderberry—*Sambucus nigra*—*SnL*, respectively) were obtained from the closed vicinity of the DS site and from the two reference sites (PH and ST areal) that have some different vegetation and soil characteristics. 

Specific aerial parts (flowers, leaves, and barks or stems, respectively) of the mentioned plants and of the plants’ roots were collected in triplicates. Additionally, *Tt* samples were collected as a mixture of flowers and leaves (*n* = 15) from 45 trees (15 per site) that were approximately 25–30 years old and approximately 30 m tall, with rich branches, and without biological or mechanical damage. 

The tree arrangement was approximately 15 m between individuals in the vicinity of the DS site, and between 15 m and 20 m at the other two sites (PH and ST). Approximately 200 g of fresh flowers and leaves were collected from each linden tree at 3–4 m above the ground. Using a stainless-steel knife, bark samples were collected at a height of 1–1.5 m from the trees’ external surface, in four different directions. For *Hp* and *SnL*, samples from the three sites were chosen based on statistical criteria. The distance between the medicinal plants was approximately 20–30 m. They were collected along with an anthropogenic pollution-expected gradient. The aerial parts or roots with obvious imperfections were removed. All vegetable samples were washed with bidistilled water. The samples of plant tissues were dried in the oven at 45–50 °C for at least 48 h until their weight was constant. A mixer grinder was used to powder the samples while preventing overheating. 

The 15 soil samples from each evaluated site were collected using a disposable plastic scalpel or a stainless-steel knife from the zone of the plants’ roots at a depth of 20 cm. A similar protocol was applied to the soil samples. The soil samples were dried for approximately 48 h at 105 °C. The dried material was triturated in a porcelain mortar to a dusty form. The sample was passed through a 100 μm sieve to ensure the uniformity of chemical composition throughout the mass of the sample, and then stored for analysis. After all of the preliminary processes, the vegetable powders and soil samples were collected in glass bottles, perfectly cleaned, and preserved until spectrometric analysis was erformed. Furthermore, the samples were weighed, digested, and analyzed using flame atomic absorption spectrometry (FAAS). The protocol for the digestion of the samples is described in [10,28]. 

The measurement of soil pH was performed using deionized water (ratio 1:2) following the NF ISO 10390/2005 norm. While stirring, approximately 10 g of soil were mixed for 30 min with 50 mL 0.1 N KCl solution. After 60 min, the mixture was filtered, and the pH was determined using a Consort P501 pH meter at 20 °C. The conductivity measurement was performed with a HACH CO150 apparatus in a solution of saturated soil.

### 2.3. Elemental Analysis

The concentrations of macroelements (Ca, Na, K, and Mg), trace elements (Cu, Zn, Mn, Fe, and Cd), and microelements in the samples was performed via FAAS [35,36,37], using a ContrAA^®^ 700 spectrometer whose main characteristics are presented in Table 1.

The concentrations of the samples are in mg/kg dry weight of the material. The performance parameters for the FAAS measurements were determined according to Barbeș et al. (2020) [10]. The domain of the concentrations (µg/L), the coefficients of correlation for the calibration curve, the limit of quantitation (LOQ), and the limit of detection (LOD) were determined for each considered element. The R^2^ values were from 0.9929 (Fe) to 0.9995 (Ca). The LOD values range was between 0.0423 mg/L (Zn) and 4.740 mg/L (Ca), and the LOQ values range was from 0.0586 mg/L (Cu) to 29.35 mg/L (K), respectively. 

Finding the elemental contents in the different aerial parts was performed using the method of curve calibration according to the absorber concentration. A similar procedure was applied to the soil samples from all analyzed sites. The procedure is described in [28]. For quality assurance, the results were checked through a triplicate analysis in concordance with NIST SRM 2709, 2710, and 2711 for soil [10,28]. The average recoveries (*n* = 5) were 88, 86, 92, 91, 83, 85, 78, 90, and 76% for Na, K, Ca, Cd, Cu, Fe, Mn, Mg, and Zn, respectively. The relative standard deviation was routinely between 0.001 and 3.6%.

### 2.4. Statistical Analyses

Apart from the basic series statistics presented in [10], the Kruskal–Wallis test [38] was performed to determine the similarities between different series collected at the study sites. More precisely, it was used to test the null hypothesis (H_0_) that two or more samples originate in the same distribution, against the hypothesis (H_1_) that at least one series has as its origin a different distribution. The groups of series to which the test was applied were: 

Test 1. The concentration of all the elements accumulated in the soil at different sites.

Test 2. The concentration of all the elements accumulated by each part of the plant, at each site.

Test 3. The concentrations of all the elements accumulated by each part of the plant, at each site.

### 2.5. Quantification of Soil Pollution Level

Soil toxicity with a number of metal elements was evaluated according to the guiding values for soils [39,40,41] that contain the reference level for the forest soils’ investigation. The geo-accumulation index (*I_geo_*), the contamination factor (*CF*), the enrichment factor (*EF*), the degree of contamination (*DC*), the pollution load index (*PLI*), the Nemerow integrated pollution index (*NIPI*), and the potential ecological risk index (*PERI*) were adopted to characterize the pollution of soil by chemical elements due to anthropogenic activities [30,31]. For a metal i, these indices are calculated by:

(1) $${\left(I_{geo}\right)}_{i}=\log_{2}\frac{C_{i}}{1.5B_{i}},$$

(2) $$CF_{i}=\frac{C_{i}}{B_{i}},$$

(3) EFi=CiCrBiBr,

where $C_{i}$—the *i*-th element concentration in the soil sample; $B_{i}$—the geochemical background value of the element *i*, $C_{r}$—the reference concentration of the element *r* in the analyzed soil; $B_{r}$—the regional background concentration [30,31,41,42,43].

The cumulated indexes are:

(4) $$DC=\overset{n}{\underset{i=1}{\sum }}CF_{i}$$

(5) $$PLI=\sqrt[{m}]{CF_{1}CF_{2}CF_{3}\ldots .CF_{m}},$$

(6) $$NIPI=\sqrt{\frac{{\left(\frac{1}{n}\sum_{i=1}^{n}CF_{i}\right)}^{2}+CF_{max}^{2}}{n}}.$$

(7) $$PERI=\overset{n}{\underset{i=1}{\sum }}(T_{i}\times CF_{i})$$

where $T_{i}$ is the toxic response of the *i*th metal [31].

### 2.6. Efficiency of Metal Accumulation in Plants

The capacity of the analyzed plant species to act as metal accumulators from the soil was evaluated, using the transfer coefficient (*TC*) and the enrichment factor (*EF*).

*TC* (or bioaccumulation factor) is computed by dividing the concentration of a metal in the aerial parts of the plant by the metal content in the soil (mg/kg). *TC* > 1 indicates the ability of the plant to absorb the metal from the soil. It is worth mentioning that not all plant species can be bio-accumulators (*TC* < 1) [16]. 

*EF* > 1 shows the existence of soil contamination and, therefore, the increased risk of the metals’ accumulation by the plants grown on those soils.

## 3. Results

### 3.1. Results of the Statistical Analysis

The results of Kruskal–Wallis test 1 provided a *p*-value = 0.9636, that means that the series of all the pollutant concentrations in the soils from the three sites issued from the same distribution. 

Table 2 and Table 3 display the results of Tests 2 and 3, respectively.

For example, in Table 2, the value of 0.9722 in the first row and the third column is the *p*-value obtained by applying the Kruskal–Wallis test to the series of the elements’ concentrations in the flowers of *SnL* collected at PH, DS, and ST. In Table 3, the value 0.5385 in the third row and the fourth column is the *p*-value computed by applying the Kruskal–Wallis test to the series of the elements’ concentrations in the stems of *SnL*, *Hp*, and *Tt* collected at ST. Since all the *p*-values in Table 2 and Table 3 are greater than 0.05 (the significance level at which this test has been performed), the null hypothesis cannot be rejected in all situations, meaning that there is no significant difference between the triples tested in each situation.

### 3.2. Ecological Risk for Analyzed Soils

The soil chemistry highlights macroelements, microelements and trace elements as different groups that can be toxic for plants and humans when their concentrations exceed set levels. As shown in Table 4, the mean concentrations of macroelements (Ca, K, Mg, and Na, respectively) varied over a range of 20.54–90.23 mg/kg (d.w.), 3.12–5.12 mg/kg (d.w.), 14.86–16.78 mg/kg (d.w.), and 0.184–0.201 mg/kg (d.w.). Additionally, the mean concentrations of microelements and trace elements in soil had the following ranges for their values: 489.9–625.8 mg/kg (d.w.)—Fe; 275.6–620.5 mg/kg (d.w.)—Mn; 2.855–35.86 mg/kg (d.w.)—Cu; 140.3–286.7 mg/kg (d.w.)—Zn, and 1.55–2.01 mg/kg (d.w.)—Cd. 

From the results obtained, calcium was the major macroelement. The decreasing series of the macroelements’ concentrations were Ca > Mg > K > Na. The concentrations of the other elements, in decreasing order, were Fe > Mn > Zn > Cu > Cd. These were comparable to the specific standard values in the Earth’s crust [38,39,40], the average European values [44], and the regional values [45], respectively (Table 5). 

The mean concentrations at DS were much lower than the Earth’s crust values for all of the elements tested, except for Cu, Zn, and Cd, for which the values recorded at the studied site were significantly higher. The average concentrations of Zn and Cd were higher than those of the Earth’s crust at PH and ST. The same was true for the comparisons with the European mean values for all the sites.

Comparisons of the average concentrations of the elements with the regional values indicate that the average concentrations Ca, Mg, Na, and Fe were below the minimum regional values, and that K was approximately at the minimum level. The concentrations of Cd were more than six times higher than the regional maximum, with Zn being in the range of the regional level, while the average concentrations of Cu were under or within the range of the regional limit values. The results obtained for microelements and trace elements indicate that Cu, Zn, and Cd recorded high values (Cu—35.86 mg/kg d.w., Zn—286.7 mg/kg d.w. in DS, and Cd—2.01 mg/kg d.w. in ST, respectively) compared to the normal values indicated in Romanian legislation (Table 6) [46]. 

The *I_geo_* values computed, taking into account the global reference and the European and local levels, are presented in Table 7. 

The indexes reported below were computed based on the global background values.

Table 8 reports on the values of the ecological risk assessment of soil in the study area (contamination factor, contamination degree, and the pollution load index).

Table 9 shows the enrichment factors for microelements and trace elements, reported for the three elements (Ca, Fe, and Mn).

The *EF*(Ca) values ranged from 0.3548 for Cu at PH, to 49.5234 for Zn at DS. *EF*(Fe) varies from 0.8742 for Cu at PH and 27.488 for Zn at DS, whereas *EF*(Mn) was between 0.1453 (for Cu at PH) and 9.6191 (for Zn at DS). There was a noticeable similarity in *EF*(Mn) behavior at PH and DS. 

The *NIPI* and *PERI* are presented in Table 10. *NIPI* was between 1.79 and 3.22, which means that the soil pollution was slight at PH, moderate at ST, and heavy at DS. *PERI* indicated a low ecological risk at PH and ST, and moderate ecological risk at DS (*PERI* = 152.84). 

### 3.3. Accumulation Factors of Metallic Species in Plants

Table 11 reports on the values of the transfer coefficients calculated for the three species analyzed in the Ciucurova area. The values of the transfer coefficients from soil to plant of the analyzed elements were above 1 for K in *SnL* samples at all three sites (PH—17.15, DS—16.80, and ST—14.52). The high potassium content in the aerial part of *SnL* could be explained by its retention by plant organs from chemical fertilizers used in fertilizing the soils within the study area. The *TC* of the other two species did not indicate high potassium concentrations in their aerial parts (leaves, flowers, or bark). For Na, the *TC* values were slightly above 1 for *Tt* at all three sites. Increased transfer coefficient values were noticeable for Cu in *Hp* and *Tt* at PH (5.01 and 5.82) and ST (1.57 and 1.48). *TC* values greater than 1 were found for Cd at DS (*Tt*—1.95) and PH site (*Hp*—1.05).

To establish the influence of soil contamination on the accumulation of elements by the selected plant species, the enrichment factor (*EF*) was calculated (Table 12). 

In our study, uncontaminated soil was considered to be soil from the PH site (a forest area located in a protected area where no specific logging activities have been conducted). DS and ST were considered to be areas with contaminated soils, for which it was possible to calculate specific enrichment factors. 

The enrichment factors had values greater than 1 in over 75% of the calculated cases. The exceptional cases were for Cu (*EF*—41.10 in DS and 3.47 in ST for *SnL*) and Cd (*EF*—12.85 in DS for *Tt*). The average concentration values of the elements (Fe, Mn, Cu, Zn, and Cd), reported as the average concentration of *SnL*, *Hp*, and *Tt*, were compared in terms of toxicity, with the permissible mean element concentration in the plants presented in Table 13 [48]. 

The results obtained from these comparisons indicated that for the three sites, Cd exceeds the non-toxic concentration values in the cases of *Hp* and *Tt*, as follows: *Hp*—0.65 mg/kg (DS), 1.63 mg/kg (PH), and 1.87 mg/kg (ST); *Tt*—0.29 mg/kg (PH), 0.34 mg/kg (ST), and 3.75 mg/kg (DS).

The transfer coefficients of macroelements, microelements, and trace elements in the plants’ nutritional medium from the three areas (PH—forest area without anthropogenic activities, ST—forest area with anthropogenic activities, and DS—tourist area in the vicinity of a forested area) varied over a large range. At DS, the *EF* for Cu accumulated by *SnL* was very high (41.10 mg/kg). Most of the *EF* for *Tt* was above 1, with the highest being that corresponding to Cd (12.85 mg/kg). 

At ST, few enrichment factors were lower than 1: that of *SnL* and *Hp* for Na and Zn, and that corresponding to *Tt* for Cu and Zn.

## 4. Discussion

With respect to the global background values, *I_geo_* showed that the analyzed soil samples were classed as soils that were moderately contaminated with Cd at all sampling points (with values between 1 and 2), and with Zn at DS and PH (values between 1.0612 and 1.9346). The soils were classed from being uncontaminated, to having moderate contamination with Cu (*I_geo_* = 0.2574) at DS, and with Zn at ST (*I_geo_* = 0.9036). These results are among the first in the study area, and a comparison with other previously recorded data was not possible. The same indicator computed with respect to the European and regional background values showed that the soils belonged to class 3 for Cd at PH and DH, and to Class 4 at DS and ST. The soils were classified as being uncontaminated with Cu, Mn, and Zn if *I_geo_* was computed based on the regional background values. The soils belonged to Class 2 for Zn at PH and DS, when the computation was performed using the global and European values, and to Class 1, for Zn at ST. 

The DC values for the soil indicated a low degree of contamination at PH (*DC* = 6.907), and a moderate degree of contamination at the other two sites (ST—8.088, and DS—11.6912, respectively). Based on the *PLI*, the soils from ST and DS belonged to the category of polluted soils due to the high content of Cd, Zn, and Cu in DS (*PLI* = 1.8915), and Cd and Zn at ST (*PLI* = 1.4467). The soil at the PH site appeared to be unpolluted (*PLI* < 1). 

Based on the *EF* values, the soils were classified into various classes, from deficient (*EF* < 2) to an extremely high enrichment in elements (Cd, Cu, Mn, and Zn). Values of *EF* between 0.5 and 1.5 indicated that the studied elements from the soil are of natural origin. In contrast, an *EF* above 1.5 shows that there is the possibility that the contamination occurred as a result of anthropogenic processes [43].

The values of *EF* for soil indicate that the soil at DS belonged to the categories of very to extremely high enrichment in Cd and Zn, with a maximum of 33.1653 (Cd) and 49.5234 (Zn). Therefore, the toxicity of Cd at the DS site was relatively high in the soil during the development of honey plants, with a destructive impact on the flora in this area. Additionally, the population consumes medicinal plants in tea infusions and bee products, which may affect their health, given that exposure to cadmium through the soil, water, air, and food leads to cancer. Its toxicity affects the skeletal, urinary, reproductive, cardiovascular, central, peripheral, and respiratory systems. The situation would not seem worrying in the case of zinc, because the concentration determination was made during the development of the melliferous plants, when an increased contribution of this element is needed in their specific metabolic processes.

The absorption of macroelements, microelements, and trace elements by plants depends on the species’ genetic properties, the content of nutrients provided by the nutrient substrate (soil), the phenophases (growth stages, development, and fruiting), the place where the plants are growing, and the climatic conditions. This result is in concordance with those from [10], where the average contents of macroelements, microelements, and trace elements found in the aerial parts (flowers, leaves, and steams, respectively, and bark) of the analyzed plants with melliferous potential (*SnL*, *Hp*, and *Tt* species) are presented. 

The *EF* values computed for plants show that elements accumulated by *SnL* and *Tt* were mainly found in flowers harvested in May and June 2019. The flowers are the most commonly used plant parts for medicinal and therapeutic purposes. Copper (whose *EF* values are the highest in *SnL* at DS and ST) is usually an essential element for growing plants. As a microelement, Cu is one of the principal elements of many enzymes of plants, but its positive actions can be limited, and it may become toxic at high concentrations. 

Another element found in high concentrations in *Tt* at DS is cadmium, which enters the environment via the atmospheric air. The presence of Cd in the atmosphere as material particles results from waste incineration and metallurgical emissions. Cadmium particles can be transported over long distances, and so the polluted area can quickly expand. Cadmium can have harmful effects on the human body after acute and long-term exposure. It is an element that accumulates in the human body with age, and elimination from the body is very difficult. 

Our findings are in concordance with the findings of other authors. 

For example, in [4], the authors emphasized the relationships between the origin of the flowers and their physical and biochemical properties. A study performed between April and September 2009 on two different zones in Romania investigated the results of translocation from the soil to different apiary products in 15 plants with melliferous potential. It was shown that the plants acted as bioaccumulators, and that contamination decreases as follows: honeybees > drones > propolis > wax > bee larvae > honey > royal jelly [49]. The authors indicated that the translocation impacts upon the safety and quality of food. A research from southeastern Poland [50] indicated the presence of heavy metal translocation, especially that of cadmium, in goldenrod and dandelion plants [50].

## 5. Conclusions

A combined approach consisting of statistical analysis and different indices was used to evaluate the degree of soil pollution and metal accumulation in plants with high melliferous potential in the area of the Babadag plateau located in the northern part of the Dobrogea region, Romania. 

The study is important for the following reasons. First, the quality of honey and its derivative products are essential for consumer health. Indeed, if plants act as accumulators, the toxic elements reach the human body by ingestion, producing different kinds of sicknesses. Secondly, this research is the first of its extent conducted for this region, where a large quantity of honey is produced. Thirdly, this approach is accessible for studying various pollutants (microelements and trace elements), and the bioaccumulation of metallic species from soil to plants. It is emphasized that using a single tool (statistics, in this case) does not always give a meaningful result. 

For a complete assessment of the pollution level in an area of analysis and, implicitly, of the toxic risk on human health through the direct or indirect consumption of different species of flora, other indicators should be used as well, such as the Estimated Daily Intakes (*EDI*), the Health Risk Index or Target Hazard Quotient (*HRI*), and the Cumulative Health Risk (*C_THQ_*). Since we noticed that different studies on the influence of soil pollution on honey quality are contradictory [49,50,51], we intend to extend the research into this direction as well. Therefore, we intend to continue the study in the following directions: (1) the analysis of the accumulation of metals in each part of *Hp*, *Tt SnL*; (2) the analysis of honey products from the same region, to determine the accumulation of toxic metals; (3) the evaluation of the risk of consumption of bee products (from the same zones).

## Figures and Tables

**Table 1:** Characteristics of the ContrAA^®^ 700 spectrometer.

	Characteristics
Light source	Xe short-arc lamp in hot-spot mode
Optical system	High-resolution double Echelle monochromator
Detector	UV-sensitive CCD (charge-coupled device) linear array
Spectral resolution	0.002–200 nm
Sensitivity	Flame: 0.015 mg/L 1% (Abs Cu-224)
Wavelength range	185–900 nm

**Table 2:** *p*-Values in the Kruskal–Wallis test for comparison of the series of the elemental concentrations in the plants components at different sites.

	Site Triplets	*SnL*	*Hp*	*Tt*
Flowers	PH—DS—ST	0.9722	0.927	0.9709
Leaves	PH—DS—ST	0.967	0.7165	0.8669
Stems	PH—DS—ST	0.9335	0.9843	0.8339
Average	PH—DS—ST	0.967	0.9468	0.7826

**Table 3:** *p*-Values in the Kruskal–Wallis test for comparison of the series of the elements concentrations in the plants components at the same site.

	PH	DS	ST	PH
Flowers *SnL*—*Hp*—*Tt*	0.8428	0.8383	0.8131	0.8428
Leaves *SnL*—*Hp*—*Tt*	0.8383	0.5578	0.7396	0.8383
Stems *SnL*—*Hp*—*Tt*	0.6928	0.3252	0.5385	0.6928
Average *SnL*—*Hp*—*Tt*	0.9518	0.7909	0.9636	0.9518

**Table 4:** Mean concentrations of elements in the soil (mg/kg d.w.)

Site	Ca	K	Mg	Na	Fe	Mn	Cu	Zn	Cd
DS	20.54 ± 1.01	3.12 ± 0.02	14.86 ± 0.34	0.201 ± 0.01	625.8 ± 4.02	275.6 ± 3.15	35.86 ± 0.14	286.7 ± 1.35	1.92 ± 0.01
PH	71.37 ± 2.01	4.05 ± 0.15	15.71 ± 0.58	0.196 ± 0.01	489.9 ± 2.03	454.1 ± 2.31	2.85 ± 0.01	156.5 ± 1.48	1.55 ± 0.02
ST	90.23 ± 3.22	5.12 ± 0.26	16.78 ± 0.47	0.184 ± 0.01	550.6 ± 3.65	620.5 ± 3.86	10.64 ± 0.10	140.3 ± 1.04	2.01 ± 0.02

**Table 5:** Element concentrations in soil for different locations (mg/kg d.w.).

	Elements		Ca	K	Mg	Na	Fe	Mn	Cu	Zn	Cd
Concentration	Earth’s crust [39,40,41]		13,700	14,000	500	6300	3800	850	20	50	0.5
	European mean value [44]		-	-	-	-	35,100	650	13	52	0.145
	Regional value [45]	Min Max	177.4 356	5 111.471	6 60	13.29 48.36	3000 4500	462.335 3046.66	14.393 109.783	57.434 423	0.02 0.24

**Table 6:** Admissible elemental concentrations in soil (mg/kg d.w.) [46].

Elements	Cd	Cu	Fe	Mn	Zn
Normal value	1.00	20.00	3000.00	900.00	100.00
Maximum threshold	5.00	250.00	4500.00	2000.00	700.00
Intervention threshold	10.00	500.00	7000.00	4000.00	1500.00

**Table 7:** Calculated values for *I_geo_*.

Reference	Site	*I_geo_*			
		Cd	Cu	Mn	Zn
Global [41]	PH	1.0473	−3.3934	−1.4894	1.0612
	DS	1.3561	0.2574	−2.2098	1.9346
	ST	1.4222	−1.4955	−1.0390	0.9036
European [43]	PH	2.8332	−2.7719	−1.1024	1.0046
	DS	3.1420	0.8789	−1.8228	1.8780
	ST	3.2081	−0.8740	−0.6520	0.8470
Regional [44]	PH	2.9907	−5.0277	−2.5349	−1.2031
	DS	3.2996	−1.3769	−3.2554	−0.3298
	ST	3.3656	−3.1298	−2.0845	−1.3608

Legend [42,43]. 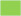 *I_geo_* ≤ 0—uncontaminated soil—Class 0; 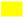 *I_geo_*: 0–1—uncontaminated to moderately contaminated soil—Class 1; 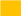 *I_geo_*: 1–2—moderately contaminated soil—Class 2; 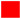 *I_geo_*: 2–3—moderately to highly contaminated soil—Class 3; 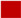 *I_geo_*: 3–4—highly contaminated soil—Class 4.

**Table 8:** Ecological risk indices.

Location	*CF*				*DC*	*PLI*
	Cd	Cu	Mn	Zn		
PH	3.1000	0.1428	0.5342	3.1300	6.9070	0.9275
DS	3.8400	1.7930	0.3242	5.7340	11.6912	1.8915
ST	4.0200	0.5320	0.7300	2.8060	8.0880	1.4467

Legend [47]. 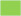 *CF* < 1—low contamination factor; *DC <* 8—low degree of contamination; *PLI* < 1—not polluted; 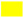 *CF:* 1–3—moderate contamination factor; 8 ≤ *DC* < 16—moderate degree of contamination; 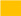 *CF:* 3–6—considerable contamination factor; 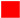 *PLI* > 1—polluted soil.

**Table 9:** Enrichment factors with respect to Ca, Fe, and Mn.

EF	Element	Location		
		PH	DS	ST
Ca	Cd	7.7055	33.1653	7.9037
	Cu	0.3548	15.4858	1.0460
	Mn	1.3279	2.8004	1.4352
	Zn	7.7800	49.5234	5.5168
Fe	Cd	18.9835	18.4084	21.9034
	Cu	0.8742	8.5954	2.8987
	Mn	3.2715	1.5543	3.9775
	Zn	19.1672	27.4880	15.2888
Mn	Cd	3.1562	6.4418	2.9953
	Cu	0.1453	3.0079	0.3964
	Mn	0.5439	0.5439	0.5439
	Zn	3.1868	9.6191	2.0908

Legend [47]. 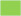 *EF* < 2—deficiency to minimal enrichment; 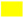 *EF:* 2–5—moderate enrichment; 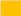 *EF:* 5–20—significant enrichment; 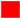 *EF*: 20–40—very high enrichment 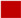 *EF* > 40—extremely high enrichment.

**Table 10:** NIPI and PERI.

Location	*NIPI*	*PERI*
PH	1.79	109.36
DS	3.22	152.84
ST	2.25	137.29

Legend [47]. 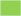 *PERI* < 150—low ecological risk; 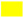 *NIPI:* 1–2—slight pollution—Class III; *PERI*: 150–300—moderate ecological risk; 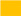 *NIPI*: 2–3—moderate pollution—Class IV; 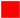 *NIPI* > 3—heavy pollution.

**Table 11:** Transfer coefficient (*TC*) for analyzed plant species.

Site	Species	Elements								
		Ca	K	Mg	Na	Fe	Mn	Cu	Zn	Cd
PH	*SnL*	0.10	17.15	0.74	0.56	0.34	0.12	0.06	0.24	0.04
	*Hp*	0.04	0.19	0.02	0.87	0.19	0.20	5.01	0.14	1.05
	*Tt*	0.09	0.57	0.21	1.04	0.23	0.09	5.82	0.20	0.19
DS	*SnL*	0.26	16.80	0.61	0.45	0.29	0.36	0.21	0.09	0.02
	*Hp*	0.09	0.21	0.01	0.69	0.25	0.28	0.62	0.15	0.34
	*Tt*	0.27	1.15	0.34	1.88	0.22	0.16	0.43	0.16	1.95
ST	*SnL*	0.10	14.52	0.76	0.45	0.32	0.10	0.06	0.21	0.06
	*Hp*	0.04	0.17	0.02	0.91	0.22	0.16	1.57	0.14	0.93
	*Tt*	0.08	0.55	0.26	1.11	0.22	0.07	1.48	0.22	0.17

**Table 12:** The enrichment factor (*EF*) for analyzed plant species.

Site	Species	Elements								
		Ca	K	Mg	Na	Fe	Mn	Cu	Zn	Cd
DS	*SnL*	0.78	0.75	0.78	0.82	1.09	1.84	41.10	0.71	0.65
	*Hp*	0.74	0.85	0.81	0.82	1.72	0.84	1.56	1.96	0.40
	*Tt*	0.89	1.54	1.50	1.85	1.26	1.14	0.93	1.41	12.85
ST	*SnL*	1.31	1.07	1.10	0.76	1.04	1.17	3.47	0.78	1.95
	*Hp*	1.58	1.16	1.53	0.99	1.32	1.11	1.17	0.89	1.15
	*Tt*	1.20	1.22	1.31	1.00	1.11	1.06	0.95	0.96	1.17

**Table 13:** Admissible mean element concentrations in plants (mg/kg d.w.) [48].

	Elements	Fe	Mn	Cu	Zn	Cd
Concentration	Non-toxic	5–250	30–300	5–30	27–150	0.05–0.2
	Toxic	-	400–1000	20–100	100–400	5–30
	Hyperaccumulation limits	10,000	10,000	1000	10,000	100

## Data Availability

Data will be available on request from the authors.
